# Treatment of Puerperal Convulsions

**Published:** 1851-05

**Authors:** R. Hull


### ARTICLE VIII

*Puerperal Convulsions* :

Dr. R. Hull of Cottage Grove, Wis., says he has recently treated a case of puerperal convulsions, which commenced two hours after delivery, by the application of fomentations to the abdomen made of smart weed, *(polyganum hydropiperoides,)* and applied as hot as could be borne. Although the case was not relieved by bleeding, enemata, and revulsives to the extremities, which had been tried for forty eight hours, there occurred but one slight convulsion after the fomentations were applied as above described.

